# Diffuse idiopathic hyperplasia of the sternocleidomastoid muscle in a child

**DOI:** 10.4103/0256-4947.57171

**Published:** 2009

**Authors:** Kamalesh Pal, Nisar Bhat, Khaled Moghazy, DK Mitra, Mohammed Hegazi

**Affiliations:** a From the Pediatric Surgery Division, Department of Surgery, King Fahad Hospital of University, Al-Khobar, Kingdom of Saudi Arabia; b From the Plastic Surgery Division, Department of Surgery, King Fahad Hospital of University, Al-Khobar, Kingdom of Saudi Arabia; c From the Department of Radiology, King Fahad Hospital of University, Al-Khobar, Kingdom of Saudi Arabia

## Abstract

Unilateral diffuse or localized enlargement of the sternocleidomastoid muscle (SCM) is an event commonly seen in infancy, and is popularly known as ‘sternocleidomastoid tumor’. The condition, which usually spontaneously resolves with or without physiotherapy, is due to a hematoma following a difficult labor. The muscle regains its elasticity and complete function. In some infants it resolves with fibromatous changes in the muscle leading to shortening, fibrosis and finally culminating in torticollis. We describe a case of idiopathic diffuse enlargement of unilateral SCM in a 12-year-old child without any functional compromise or torticollis. The histopathological and clinical characteristics differentiating it from more commonly described sternocleidomastoid tumor or fibromatosis coli are described. We believe this is the first case report of idiopathic hyperplasia of SCM.

Sternocleidomastoid swelling is commonly encountered in infancy as ‘fibromatosis coli’ or ‘immature infantile tumor’ following a hematoma due to difficult labor.^1^ We report a case of spontaneous unilateral diffuse enlargement of the left sternocleidomastoid muscle (SCM) giving rise to progressive neck swelling in a 12-year-old child. We discuss the unique clinical and histopathological characteristics of this swelling, which can be classified as idiopathic hyperplasia of the SCM.

## CASE

**Figure 1:**
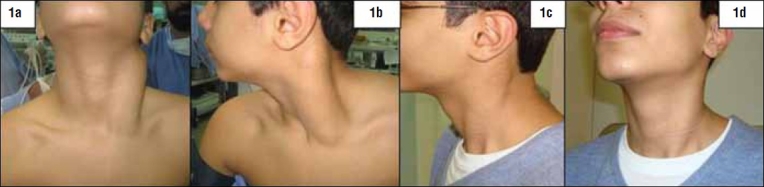
Diffuse enlargement of left sternocleidomastoid muscle without torticollis. Preoperative (1a and 1b); postoperative (1c and 1d) status.

**Figure 2:**
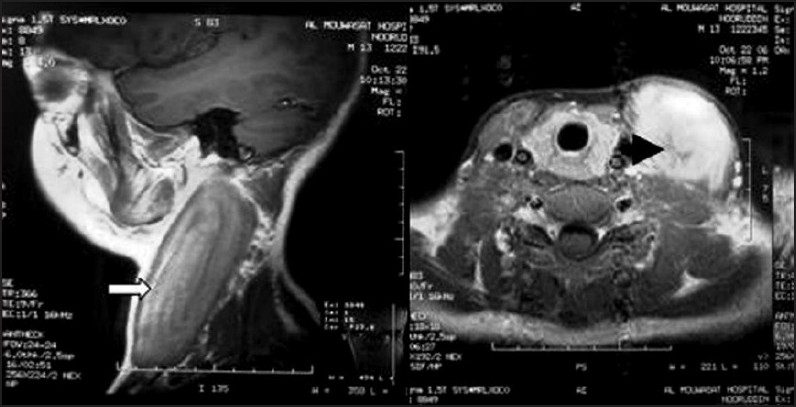
MRI of neck showing diffuse enlargement of left SCM (isointense on T1 (open arrow) and mildly hyperintense on T2 and enhancing after IV contrast (block arrow).

A 12-year-old healthy boy presented with spontaneously developed progressive painless swelling of the left sternocleidomastoid muscle in the neck extending linearly from the mastoid process to the sternoclavicular joint along the of same side for 3 years (Figures 1a and 1b). The clavicular head of the muscle did not share the hypertrophy. There were no associated symptoms of fever, restriction of neck movement or weight loss. Workup at another center revealed a fusiform hypoechoic mass along the left SCM on ultrasonography. MRI scan revealed a 12×5×4 cm diffuse hypointense mass on T1-weighted image replacing the left SCM, which was mildly hyperintense on T2 and showed enhancement on IV contrast (Figure 2). There was no enlargement of the cervical lymph nodes or perimysial extension. The parotid, submandibular and thyroid gland appeared normal. Fine needle aspiration biopsy revealed skeletal muscle with degenerative and reactive cellular changes, possibly myositis. Incisional biopsy also reported the histopathology to be consistent with focal myositis (pseudotumor). At presentation to our hospital, the swelling had increased to a size of 15×6×5 cm^3^. There was no associated restriction of neck movements or torticollis. On palpation the entire swelling was firm, and nontender with overlying normal skin and no fixation to underlying structures. The forced contraction maneuver for left SCM would make the swelling taut. There was no significant cervical lymphadenopathy.

**Figure 3:**
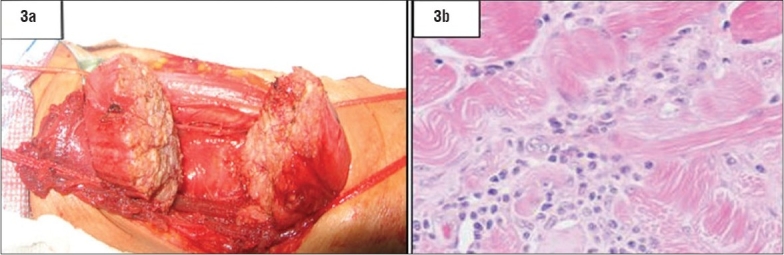
Gross (3a) and histopathology (3b) of excised left SCM showing hyperplasia and chronic inflammatory cell infiltration. (Histopathology is not available)

Due to the progressive increase in size over the previous 3 years, as well as cosmetic issues and the unexplained pathology, the father sought surgical advice and agreed to excision of the left sternocleidomastoid swelling under general anaesthesia. A diagnostic and therapeutic excision of the swollen SCM was carried out through a lateral collar incision overlying the most prominent part of the swelling. Histopathological examination of the specimen revealed skeletal muscle hyperplasia with focal interstitial predominantly chronic inflammatory cellular infiltrate (Figure 3), focal endomysial connective tissue proliferation and a fibroblastic reaction together with degenerative and reactive skeletal muscle fiber changes. No atypia was detected. The histopathological diagnosis was that of a nonspecific proliferative myositis of the muscle.

The child had regular follow-up to observe for any future changes in the contralateral SCM or other neck muscles. At 1-year follow-up he remained well without any recurrence or deformity (Figures 1c and 1d). The child compensated for neck movement by using strap muscles and anterior fibers of the trapezious muscle

## DISCUSSION

Unilateral swelling in the neck of a child evokes a list of differentials, including cervical lymphadenopathy (commonest), branchial cyst, cystic hygroma, dermoid cyst, thyroid lobe enlargement, pharyngeal pouch, cold abscess and sternocleidomastoid tumor (in infants). Clinical examination of the neck helps in differentiating the site and nature of the swelling to a large extent.

Diffuse enlargement of SCM in infancy is known to occur at around 3 weeks to 3 months of age. The cause is unknown although ‘idiopathic intrauterine embryopathy’^2^ and intrauterine positional disorder with development of ‘sternocleidomastoid compartment syndrome’^3^ has been postulated for its genesis. The basic change in histopathology is fibrous replacement of muscle bundles. A process of endomysial fibrosis involves deposition of collagen and fibroblasts around individual muscle fibers that undergo atrophy.^2^,^4^ The natural history of untreated sternomastoid fibrosis is complete resolution in 50% to 70% of patients at 6 months of age. In less than 10% of cases SCM shortening persists beyond 1 year of age.^5^

Shortening of SCM leads to ipsillateral torticollis which on longterm can lead to hemifacial hypoplasia (due to lack of muscular traction, the mandible and maxilla undergo hypoplasia), plagiocephaly in infancy and compensatory scoliosis in an older child.

In our case, there was spontaneous enlargement of the left SCM in its entire length, which was supple, and without shortening or lengthening failure, so there was no torticollis or limitations in rotation of the neck. The lack of symptoms was so distinct that the decision of excision was taken only due to progressively increasing swelling of the entire SCM and the uncertain nature of the pathology, in addition to concern for cosmesis. Debulking was not considered an option due to fear of recurrence. Post excision histopathological examination revealed hyperplasia of the muscle spindles with chronic inflammatory infiltrate with no atypia or granulomas. There was no fibromatous change seen in the muscle, thus differentiating it from sternomastoid tumor or fibromatosis coli.

Some studies^6^–^10^ have shown that the use of progesterone, anti-estrogens, nonsteroidal antinflammatory drugs, warfarin, vitamin K and ascorbate in various combinations bring some response in halting the fibromatosis, mostly in desmoid tumors, but not so in infantile fibromatosis coli or sternocleidomastoid tumors. Therefore, the reasonable approach for treatment of even fibromatosis of the head and neck is complete surgical excision to avoid recurrences.^11^ However, in our case there was no fibromatous change (rather there was hyperplasia of muscle) seen in the muscle, thus differentiating it from sternocleidomastoid pseudotumor or fibromatosis coli and precluding the option of anti-inflammatory drug therapy.

Idiopathic hyperplasia of SCM, as we have coined this entity in our case, is a nonfibromatous enlargement of the SCM, spontaneous in onset, histologically characterized by chronic inflammatory cell infiltrate limited to the SCM only and associated with no shortening or lengthening failure or torticollis. Muscle function is essentially preserved. Further study is needed to understand the natural course of this entity.

## References

[1] Cheng JC, Tang SP, Chen TM (1999). Sternocleidomastoid pseudotumor and congenital muscular torticollis in infants: A prospective study of 510 cases. J Pediatr.

[2] Jones PG (1967). Torticollis in Infancy and childhood.

[3] Hirschl RB, Spitz L, Coran A (1995). Sternocleidomastoid torticollis. Rob & Smith's Operative Surgery. Pediatric Surgery.

[4] Middleton DS (1930). The pathology of congenital torticollis. Br J Surg.

[5] de Chalain TM, Katz A (1992). Idiopathic muscular torticollis in children: The Cape Town experience. Br J Plast Surg.

[6] Waddell WR, Gerner RE, Reich MP (1983). Nonsteroidal anti-inflammatory drugs and tamoxifen for desmoid tumors and carcinoma of the stomach. J Surg Oncol.

[7] Waddell WR (1975). Treatment of intra-abdominal and abdominal wall desmoid tumor with drugs that affect the metabolism of cyclic 3′,5′-adenosine monophosphate. Ann Surg.

[8] Waddell WR, Gerner RE (1980). Indomethacin and ascorbate inhibit desmoid tumors. J Surg Oncol.

[9] Waddell WR, Kirsch WM (1991). Testolactone, sulindac, warfarin, and vitamin K1 for unresectable desmoid tumors. Am J Surg.

[10] Sauven P (1982). Musculo-aponeurotic fibromatosis treated by surgery and testolactone. J R Soc Med.

[11] Das Gupta TK, Brasfield RD, O'Hara J (1969). Extra-abdominal desmoids: a clinicopathologic study. Ann Surg.

